# Heterogeneous Pattern of Selective Pressure for PRRT2 in Human Populations, but No Association with Autism Spectrum Disorders

**DOI:** 10.1371/journal.pone.0088600

**Published:** 2014-03-03

**Authors:** Guillaume Huguet, Caroline Nava, Nathalie Lemière, Etienne Patin, Guillaume Laval, Elodie Ey, Alexis Brice, Marion Leboyer, Pierre Szepetowski, Christopher Gillberg, Christel Depienne, Richard Delorme, Thomas Bourgeron

**Affiliations:** 1 Human Genetics and Cognitive Functions, Institut Pasteur, Paris, France; 2 CNRS URA 2182 ‘Genes, synapses and cognition’, Institut Pasteur, Paris, France; 3 University Denis Diderot Paris 7, Paris, France; 4 INSERM, U975—CRICM, Institut du cerveau et de la moelle épinière (ICM), Hôpital Pitié-Salpêtrière, Paris, France; 5 CNRS 7225—CRICM, Hôpital Pitié-Salpêtrière, Paris, France; 6 Université Pierre et Marie Curie-Paris-6 (UPMC), UMR_S 975, Paris, France; 7 Département de Génétique et de Cytogénétique, Unité fonctionnelle de génétique clinique, AP-HP, Hôpital Pitié-Salpêtrière, Paris, France; 8 Unit of Human Evolutionary Genetics, Institut Pasteur, Paris, France; 9 Département de Génétique et de Cytogénétique, Unité fonctionnelle de neurogénétique moléculaire et cellulaire, AP-HP, Hôpital Pitié-Salpêtrière, Paris, France; 10 INSERM, U955, Psychiatry Genetic team, Creteil, France; 11 Fondation FondaMental, Créteil, France; 12 INSERM, UMR_S901, Marseille, France; 13 Aix-Marseille University, Marseille, France; 14 Mediterranean Institute of Neurobiology (INMED), Marseille, France; 15 Gillberg Neuropsychiatry Centre, University of Gothenburg, Göteborg, Sweden; 16 Institute of Neuroscience and Physiology, Department of Pharmacology, Gothenburg University, Gothenburg, Sweden; 17 Institute of Child Health, University College London, London, United Kingdom; 18 Assistance Publique-Hôpitaux de Paris, Robert Debré Hospital, Department of Child and Adolescent Psychiatry, Paris, France; Oslo University Hospital, Norway

## Abstract

Inherited and *de novo* genomic imbalances at chromosome 16p11.2 are associated with autism spectrum disorders (ASD), but the causative genes remain unknown. Among the genes located in this region, *PRRT2* codes for a member of the synaptic SNARE complex that allows the release of synaptic vesicles. *PRRT2* is a candidate gene for ASD since homozygote mutations are associated with intellectual disability and heterozygote mutations cause benign infantile seizures, paroxysmal dyskinesia, or hemiplegic migraine. Here, we explored the contribution of *PRRT2* mutations in ASD by screening its coding part in a large sample of 1578 individuals including 431 individuals with ASD, 186 controls and 961 individuals from the human genome Diversity Panel. We detected 24 nonsynonymous variants, 1 frameshift (A217PfsX8) and 1 in-frame deletion of 6 bp (p.A361_P362del). The frameshift mutation was observed in a control with no history of neurological or psychiatric disorders. The p.A361_P362del was observed in two individuals with autism from sub-Saharan African origin. Overall, the frequency of *PRRT2* deleterious variants was not different between individuals with ASD and controls. Remarkably, *PRRT2* displays a highly significant excess of nonsynonymous (pN) *vs* synonymous (pS) mutations in Asia (pN/pS = 4.85) and Europe (pN/pS = 1.62) compared with Africa (pN/pS = 0.26; Asia *vs* Africa: P = 0.000087; Europe *vs* Africa P = 0.00035; Europe *vs* Asia P = P = 0.084). We also showed that whole genome amplification performed through rolling cycle amplification could artificially introduce the A217PfsX8 mutation indicating that this technology should not be performed prior to *PRRT2* mutation screening. In summary, our results do not support a role for *PRRT2* coding sequence variants in ASD, but provide an ascertainment of its genetic variability in worldwide populations that should help researchers and clinicians to better investigate the role of *PRRT2* in human diseases.

## Introduction

Autism Spectrum Disorders (ASD) are characterized by impairments in reciprocal social communication, and repetitive, stereotyped and ritualistic behaviors [1]. ASD include autism, Asperger syndrome and pervasive developmental disorder not otherwise specified (PDD-NOS). The prevalence of ASD overall is about 1/100, but closer to 1/300 for autism with intellectual disability (ID) [2]. The susceptibility genes to ASD remain largely unknown, but mutations affecting genes such as *NLGN3/4X*, *SHANK2/3*, *NRXN1* and *CNTNAP2* were shown to alter synaptic function and increase the risk for ASD [3]. Several large-scale studies have reported an enrichment of copy number variants (CNVs) in individuals with ASD [4], [5], suggesting that gene dosage plays a key role in the susceptibility to ASD [6]. Among the recurrent genomic alterations associated with ASD, *de novo* or inherited submicroscopic microdeletion/duplication at 16p11.2 have been associated with a variety of developmental/neuropsychiatric disorders including ASD, intellectual disability (ID), schizophrenia, or bipolar disorders, but also with body mass index [7]–[14]. A meta-analysis of 6 studies including more than 2000 individuals and 30000 controls provided strong support for the role of recurrent 16p11.2 genomic imbalances as risk factors for ASD (duplication OR:20.7 and P = 1.9×10^−7^; deletion OR:38.7 and 2.3×10^−13^) [13]. The deleted/duplicated region at 16p11.2 spans 500–600 kb and is flanked by large and highly similar 147 kb low copy repeats with >99% homology, predisposing to unequal crossing-over during meiosis [10]–[14]. Among the 27 genes located within the interval, Kumar *et al.* (2009) screened for rare variations in 8 candidate genes selected for their function and expression in the brain (*ALDOA*, *DOC2A*, *HIRIP3*, *MAPK3*, *MAZ*, *PPP4C*, *SEZ6L2* and *TAOK2*). None of them showed a significant association with ASD at the exception of *SEZ6L2* for which a suggestive association was detected [15], but not confirmed [16]. Smaller deletions at 16p11.2 were identified in individuals with ASD narrowing the critical region and including the *PRRT2* gene [8], [9]. *PRRT2* codes for a membrane protein that interacts with SNAP25, a protein from the synaptic vesicles release machinery (the SNARE complex). *PRRT2* is a compelling candidate gene for ASD and other psychaitric diseases since a homozygote frameshift mutation (A217PfsX8) was shown to segregate with non-syndromic ID in one consanguineous family with 9 affected individuals [17]. Furthermore numerous heterozygote frameshift, nonsense and missense *PRRT2* mutations (including the recurrent frameshift A217PfsX8) were identified in individuals and families with benign infantile seizures (BIS), paroxysmal kinesigenic dyskinesia (PKD), hemiplegic migraine (HM), or episodic ataxia, variably associated (*e.g.* the ICCA syndrome that associates BIS with PKD) [18]–[21]. Following these results, we compared the frequency of deleterious mutations in 431 individuals with ASD and 186 controls. In addition, we ascertained the genetic variability of *PRRT2* in a sample of 961 individuals from worldwide populations.

## Materials and Methods

### Individuals and control samples

Mutation screening of *PRRT2* was performed in individuals with ASD recruited by the PARIS (Paris Autism Research International Sibpair) study at specialized clinical neuropsychiatric centers disposed in France and Sweden (Table S1). Diagnosis was based on comprehensive clinical evaluation by experienced clinicians using DSM IV-TR criteria; most individuals were assessed with the Autism Diagnostic Interview-Revised (ADI-R) and some of them also with the Autism Diagnostic Observation Scale (ADOS). In Sweden, in some cases, the Diagnostic Interview for Social and Communication Disorders (DISCO-10) was used instead of the ADI-R. Cases were included only after a thorough clinical evaluation, including psychiatric and neuropsychological examination, standard karyotyping, and fragile-X testing, as well as brain imaging and EEG whenever possible. For comparison between cases and controls, only individuals from European descent were considered (Table S2). The Human Genome Diversity Panel (HGDP) is a collection of 961 individuals from worldwide populations [22]. This study was approved by the local Institutional Review Board (IRB). The local IRB are the Comités de Protection des Personnes Île-de-France VI Sis Hôpital Pitié-Salpêtrière 75013 PARIS for France and the Sahlgrenska Academy Ethics committee, University of Gothenburg for Sweden. For all probands written inform consent was signed by the individuals or parents or the legal representative.

### Mutation screening of PRRT2

We obtained DNA of 431 unrelated individuals with ASD, 180 controls and 961 individuals without known clinical status from the Human Genome Diversity Panel (HGDP) from blood leukocytes or B-lymphoblastoid cell lines, and was extracted with phenol-chloroform (Table S1, S2). All coding exons of *PRRT2* were screened by direct sequencing of the PCR products (Table S3). The 3 coding exons of PRRT2 (NP_660282.2) and one supplementary exon present in the splicing isoforms (Q7Z6L0-1, Q7Z6L0-2, Q7Z6L0-3) were sequenced. For the amplicon of exon 2, that contains the frameshift mutation A217PfsX8, we screened an additional sample of 295 individuals with ASD and 92 controls. For all exons, the amplification of 20 ng of DNA template was performed using a standard PCR protocol with the FastStart Taq polymerase (Roche): 95°C for 15 min, followed by 35 cycles of 95°C for 30 s, 55 to 64°C (depending on the Tm) for 30 s, 72°C for 30 s to 1 min (depending on the product size), with a final cycle at 72°C for 10 min. Direct sequencing of the PCR products was performed using the BigDye Terminator Cycle V3.1 Sequencing Kit, and an ABI PRISM genetic analyzer (Applied Biosystems). For all nonsynonymous variations, the genotype was confirmed by sequencing an independent PCR product. Because of the low quantity of DNA for 3 individuals with ASD, we used whole genome amplification (WGA), using two commercially available Multiple Displacement Amplification kits. For the GenomiPhi DNA Amplification Kit (Amersham Pharmacia, Uppsala, Sweden), 25 ng of genomic DNA (in 2.5 µl) were mixed with 22.5 µl of GenomiPhi sample buffer. For the Repli-G Whole Genome Amplification kit (Qiagen Sciences Inc. Germantown, MD), 25 ng of genomic material were diluted in TE to a final volume of 2.5 µl. For comparison with the exome variant server, we verified that the coverage of the *PRRT2* exons was satisfactory (92% of the coding region had a coverage >20; Figure S1).

### Statistical analyses and software

The significance of differences in *PRRT2* variants in individuals and controls was determined by a two-sided Fisher's exact test on a two-by-two contingency table. PolyPhen-2 (http://psort.hgc.jp) and PSORT (http://genetics.bwh.harvard.edu/pph2) were used to predict the functional impact of amino acid substitutions and trans-membrane segments, respectively. To estimate the selective pressure on PRRT2 in different populations (pN/pS), the number of synonymous SNPs or nonsynonymous SNPs was divided by the total number of synonymous or nonsynonymous positions in PRRT2. The number of synonymous and nonsynonymous sites of PRRT2 were calculated by DnaSP v5 [23].

## Results

**Figure 1:**
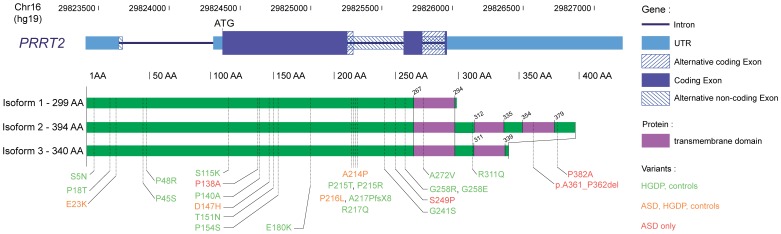
*PRRT2* coding variants identified in this study. Schematic diagram of the *PRRT2* gene and of the three PRRT2 protein isoforms. Mutation identified in this study are indicated in green (controls and HGDP), orange (controls, HGDP, and patients) and red (patients only).

We sequenced all coding exons of *PRRT2* in a large cohort of 1578 individuals including 431 individuals with ASD, 186 controls and 961 individuals from the HGDP [22]. None of the patients tested were deleted or duplicated for the 16p11.2 locus. Overall, we identified 24 nonsynonymous variants, 1 frameshift A217PfsX8 and 1 in-frame deletion of 6 bp (Del 361A-362P) (Table 1, Figure 1, Table S4 and S5). Among those, 18 were not reported before (S5N, P18T, P48R, S115K, P140A, T151N, P154S, E180K, A214P, P215T, G241S, S249P, G258E, G258R, A272V, R311Q, p.A361_P362del), 1 (A217PfsX8) was repeatedly found in individuals with BIS, PDK and/or HM as well as in unaffected carriers, and 7 (E23K, P45S, P138A, D147H, P215R, P216L, R217Q) were previously observed in the general population (listed in Exome Variant Server, dbSNP or in the 1000 genomes project, Table S6). After stratification for European ancestry, we did not observe significant enrichment of coding sequence variants in the patient sample compared with controls (Table 1). This absence of enrichment was also observed when only variants predicted as deleterious were taken into account.

**Table 1:** *PRRT2* coding variants identified in individuals with ASD and controls.

Variants	dbSNP/EVS/1KG	Inheritance	Ethnicity	Polyphen2	ASD (All; European ancestry)	Controls from European ancestry	Odds ratio [.95 CI]^c^	P value^c^
E23K^a^	1KG_16_29824442	ND	2 Europe	Benign	2/431; 2/261	0/186		
P138A^a^	rs79182085	ND	1 Asia	Benign	1/431; 0/261	0/186		
D147H^a^	rs79568162	Maternal	1 Asia	Benign	1/431; 0/261	0/186		
A214P^a^	-	4 Maternal	1 Asia, 1 Europe, 2 Africa	Damaging	4/726; 1/474	0/272		
P216L^a^	rs76335820	2 Maternal, 1 Paternal, 5 ND	6 Europe, 1 mixed, 1 ND	Damaging	8/726; 6/474	8/272		
A217PfsX8	-	ND	1 Europe	Damaging	0/726; 0/474	1/272		
S249P	-	ND	1 mixed	Damaging	1/726; 0/474	0/272		
p.A361_P362del	-	2 Maternal	2 Sub Saharan Africa	Damaging^b^	2/431; 0/261	0/186		
P382A	-	Maternal	1 mixed	Damaging	1/431; 0/261	0/186		
All variants					17/431; 6/261	6/186	0.71 [0.23–2.24]	0.77
All damaging					13/431; 3/261	6/186	0.36 [0.09–1.44]	0.17

a Variants observed in the human genome diversity panel.

b The p.A361_P362del was considered as probably damaging since it affects conserved amino acids of PRRT2.

c Odds ratio, confidence intervals (CI) and P values were calculated only for populations from European ancestry using a 2-tailed Fisher exact test.

In the patient sample, we were able to ascertain the inheritance of the variants for 10 families and found that all mutations were inherited. Interestingly, the majority of the mutations were inherited from the mother (9/10; P = 0.012). In two individuals with ASD from sub-Saharan African origin, a 6 bp in-frame deletion (p.A361_P362del) was identified. The variant was transmitted by unaffected mothers and never observed in any individuals from our study and from other *PRRT2* mutation screening or in the 1000 genomes (>2000 controls). The genomes of these two individuals were investigated using the Illumina 1M Duo SNP array. Using Identity by state (IBS) analysis, we showed that these two individuals were not relatives (Table S7), but were clustered with individuals from South Africa and Kenya (Fig. S2). This *PRRT2* deletion of 2 conserved amino acids occurs in a predicted transmembrane domain of a specific isoform of *PRRT2* (Figure 1). *In silico* prediction using the PSORT software suggests however that the deletion does not disrupt the transmembrane domain of this isoform.

In the control sample, we identified one individual carrying the frameshift mutation A217PfsX8 already described as causing autosomal dominant ICCA, BIS, PKD, HM or epilepsy) [18]–[21]. This control individual had no history of psychiatric and neurological disorders. To further explore the prevalence of A217PfsX8, we screened an additional sample of 295 individuals with ASD and 92 controls. Overall, A217PfsX8 was observed only in the control described above and not detected in any of the other 277 controls, 726 individuals with ASD and in any of the 961 individuals from the HGDP.

**Figure 2:**
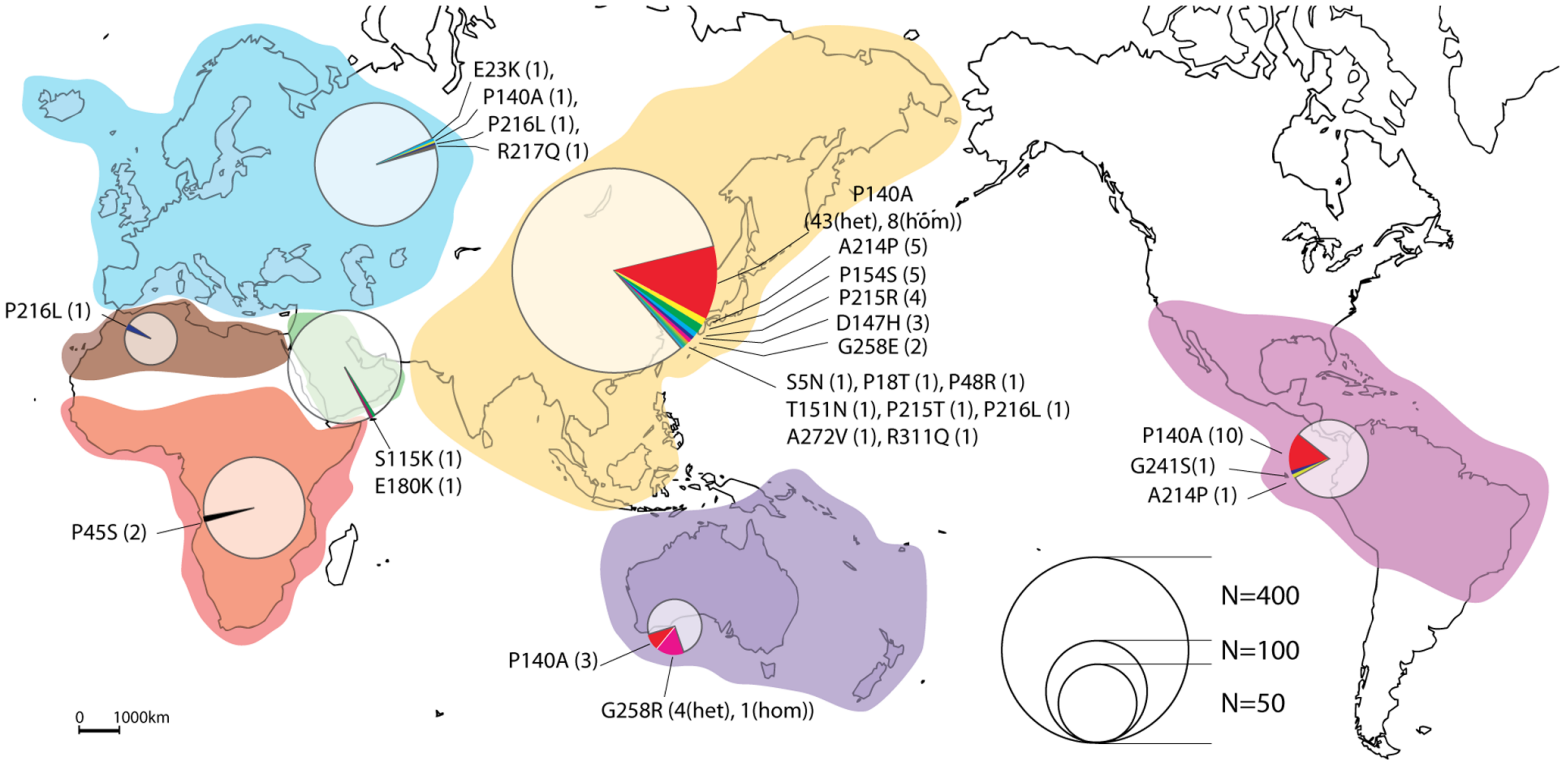
*PRRT2* variants identified in individuals from worldwide populations. A total of 961 individuals from the human genome diversity panel (HGDP) were sequenced for all *PRRT2* exons. The diameter of each circle is proportional to the number of individuals who were sequenced for *PRRT2*.

We used a panel of DNA from individuals originated from worldwide populations (Figure 2, Table S4 and S5). We could identify 21 nonsynonymous *PRRT2* variants and trace the possible origin of them. For example, the predicted damaging P140A is a relatively frequent polymorphism present in Asia (7%), Oceania (5%), in Native American (7%), but more rare in Europe (allele frequency <1%). The A214P variant was observed in Asian (allele frequency <1%) and Native American populations. The P216L was observed in North Africa (allele frequency 1,7%), Europe (<1%) and Asia (<1%). Interestingly, we observed a higher ratio of nonsynonymous *vs* synonymous variants in Asia compared with Africa. In Asia, we found only 1 synonymous variant (E127E) carried by a single individual (allelic frequency of synonymous variants: 1/878 = 0.12%) and 15 nonsynonymous variants (S5N, P18T, P48R, P140A, D147H, T151N, P154S, A214P, P215T, P215R, P216L, G258E, A272V, R311Q) carried by 78 individuals (allelic frequency of nonsynonymous variants: 86/878 = 9,8%). In contrast, in Africa, we found 3 synonymous variants (P215P, L251L, C276C) carried by 7 individuals (allelic frequency of synonymous variants: 8/216 = 3.7%) and only 1 nonsynonymous variant (P45S) carried by 2 individuals (allelic frequency of nonsynonymous variants: 2/216 = 0.92%).

**Figure 3:**
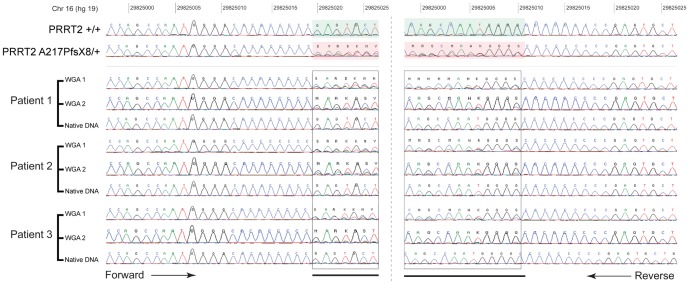
Genetic variability in Europe and Africa for all genes located within the 16p11.2 deletion. A. Nonsynonymous mutations per nonsynonymous sites (pN) and synonymous mutations per synonymous sites (pS) were estimated using the data from 4300 individuals from European ancestry and 2012 individuals from African ancestry available at the Exome Variant Server (http://evs.gs.washington.edu/EVS/). The horizontal lanes correspond to the means of pN and pS for the 27 genes. Difference of pN/pS between Europe and Africa were calculated using a 2-tailed Fisher exact test and the −Log_10_ P value is indicated. B. Plot of the −Log_10_ P values obtained for the difference between the pN/pS ratio in Africa and in Europe in relation the ratio (Europe pN/pS)/(Africa pN/pS).

To evaluate selective constraints on PRRT2 in the different populations, we estimated pN/pS the ratio of nonsynonymous (pN) to synonymous polymorphism (pS) in each population sample (Table 2). Usually, most nonsynonymous changes would be expected to be eliminated by purifying selection, but under certain conditions positive Darwinian selection may lead to their retention [24]. Investigating the number of synonymous and non-synonymous substitutions therefore provides information about the degree of selection operating on a gene. A pN/pS ratio greater than one implies positive Darwinian selection; less than one implies purifying (stabilizing) selection; and a ratio of one indicates neutral (i.e. no) selection [25]. The calculation of the pN/pS ratio in the different populations revealed sign of positive Darwinian selective pressure on PRRT2 in Asia (pN/pS = 4.85) compared to Africa (pN/pS = 0.17; Fischer exact test P = 0.000087). For Europe (pN/pS>1.5), there was a trend for higher nonsynonymous mutations compared to Africa (Fischer exact test P = 0.057). We therefore tested if this difference was also observed between American European and American African populations available in the Exome Variant Server database (Table 2). Using this large dataset of 4300 individuals from European ancestry and 2012 individuals from African ancestry, we could confirm that there was a significant increase of nonsynonymous variants in Europe (pN/pS = 1.62) compared to Africa (pN/pS = 0.26) (Fischer exact test P = 0.00035). Finally, we observed a trend for higher frequency of nonsynonymous variants in Asia compared to Europe (Fischer exact test P = 0.084). As expected, there was no difference between populations from the HGDP and those from EVS (Africa HGDP *vs* Africa EVS P = 0.67; Europe HGDP *vs* Europe EVS P = 1). In order to test whether this difference in selective pressure was specific to *PRRT2*, we used the data from EVS and extended our pN/pS calculation to all genes included in the 16p11.2 deletion (Figure 3; Table S8). We observed that the majority of the genes had a very similar pN/pS in Europe and Africa. Only C16orf92 showed a higher pN/pS in Africa compared to Europe. In contrast, ASPHD1, YPEL3, MAPK3 and PRRT2 showed a higher pN/pS in Europe compared to Africa. However, *PRRT2* was the only gene with a pN/pS significantly different in Europe and Africa after correction for multiple testing.

**Table 2:** Synonymous and nonsynonymous variations of *PRRT2* in worldwide populations.

Ethnic	Nonsynonymous variants	Synonymous variants	pN (nonsynonymous variants/nonsynonymous sites)^a^	pS (synonymous variants/synonymous sites)^a^	pN/pS
EVS Europe (N = 4300)	14	3	0.021	0.013	1.62
EVS African-American (N = 2012)	6	8	0.009	0.035	0.26
HGDP Asia (N = 439)	14	1	0.021	0.004	4.85
HGDP Africa (N = 108)	1	2	0.001	0.009	0.17
HGDP Europe (N = 159)	4	0	0.006	0.000	>1.5^b^

a Number of nonsynonymous and synonymous sites for PRRT2 are 668.5 and 231.5, respectively.

b For the calculation of the pN/pS for Europe, the value of pS was set to 0.004 (the minimum of 1 synonymous variant observed in 159 individuals).

**Figure 4:**
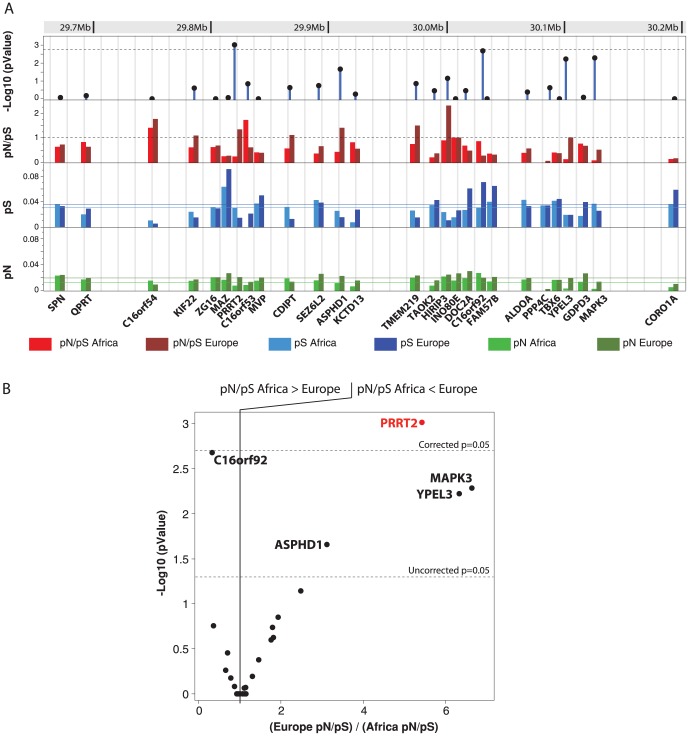
Chromatograms of the *PRRT2* A217PfsX8 mutation before and after whole genome amplification. The chromatograms of *PRRT2* sequence before (Native DNA) and after whole genome amplification of the DNA from three independent patients using two different protocols GenomiPhi DNA Amplification Kit (WGA-1) or Repli-G Whole Genome Amplification kit (WGA-2). The mutation was not present in the native DNA, but was detected after whole genome amplification.

During the screening of the recurrent frameshift mutation ‘A217PfsX8’, we used whole genome amplified DNA for three individuals with ASD. Strikingly, we observed the A217PfsX8 mutation in these three samples for which the DNA was amplified using rolling circle amplification protocol (Genomiph from GEHealthcare) (Figure 4). We obtained the native DNA from these three individuals and found that they were actually not carrying the A217PfsX8 mutation. We then re-amplified the native DNA from these three individuals using another RCA protocol (REPLI-g Mini method from QIAGEN). After direct sequencing of the PCR products, the A217PfsX8 frameshift mutation was again present. Based on these results, we strongly advise that *PRRT2* mutation screening should not be performed on DNA previously amplified through RCA.

## Discussion

Both deletions and duplications at 16p11.2 increase the risk for ASD, but the genes and the mechanisms involved remain largely unknown. Whereas several other strong candidate genes including the *KCTD13* gene are located within the deletion and can be considered possible driver(s) as well [26], *PRRT2* was indeed a very compelling candidate. Homozygous PRRT2 mutations were associated with clinical features also observed in a subset of individuals with ASD such as intellectual disability and epilepsy. *PRRT2* encodes a putative membrane protein, localized at the synaptic membrane and interacting with SNAP25, a member of the SNARE protein complex involved in the release of synaptic vesicles. Our study was designed to identify new coding variants of *PRRT2* in a relatively large sample of individuals with ASD and to ascertain the genetic variability of the gene in worldwide populations.

We could not find any deleterious *PRRT2* mutation enrichment in individuals with ASD compared with controls. None of the patients tested were deleted or duplicated for the 16p11.2 locus. In addition, we did not find causative *PRRT2* mutations previously associated with neurological diseases such as PDK, ICCA, epilepsy or migraine in our cohort of patients. We observed a significant enrichment of maternally transmitted mutations in the individuals with ASD (9/10 transmitted by the mother). However, this apparent disequilibrium might have occurred by chance since to our knowledge, no transmission disequilibrium was reported for *PRRT2* mutations in other neurological diseases. Taken together, these results indicate that mutations in *PRRT2* are not a frequent cause of autism. Our study cannot however exclude that, in individuals with 16p11.2 deletions, haploinsufficiency of *PRRT2* could increase the risk of ASD.

Secondly, we detected the presence of the deleterious truncating mutation A217PfsX8 in a control (of note the DNA of this individual was not whole genome amplified). This man had no psychiatric diseases and, to his knowledge, no history of any psychiatric disorders in his first and second degree relatives (parents and grand parents). This individual had no diagnostic for migraine and epilepsy, but was unavailable for further clinical explorations in order to detect mild signs of neurological problems. Similar incomplete penetrance of the A217PfsX8 mutation was observed in carriers from families with ICCA syndrome [21]. Based on the literature, the penetrance of the A217PfsX8 mutation was estimated to 94% [27]. It remains unknown if additional genetic variants could act as suppressors in the unaffected individuals carrying the A217PfsX8 mutation.

Thirdly, we ascertained the genetic variability of *PRRT2* in worldwide populations. We identified 21 novel nonsynonymous variants including 17 never reported before and 4 already listed in the nucleotide variant database (Table S6). Despite the relatively large number of chromosomes tested and the population diversity, we did not observe any truncating mutations. Most of the nonsynonymous variants are restricted to one population (D147H, P154S, E180K, P215R, G241S, G258E and G258R) or to closely related populations (A214P and P216L). One variant P140A was frequently observed in different populations with a shared genetic origin (Asia, Native American and Oceania). Overall, the low rate (<1%) of nonsynonymous mutation indicates that *PRRT2* is under strong selective pressure. Nevertheless, our results also revealed a heterogenous pattern of selective pressure acting on *PRRT2* in human populations. The low pN/pS ratio in Africa indicates *PRRT2* is under the usual purifying selection (as for the majority of the genome), whereas the pN/pS ratio in Asia and Europe is higher than expected and consistent with positive Darwinian selection [28]. To date, we have no explanation for this observation. Within the 16p11.2 deletion, *PRRT2* seems to be the gene with the higher difference of selective pressure between Europe and Africa, but addition samples should be screened to ascertain if this difference is specific to *PRRT2* or is also true for other genes within the deletion. Considering this heterogeneous pattern of selective pressure, it would be interesting to see if there is a higher prevalence of *PRRT2* related disease in Asia and Europe compared to Africa. It would also be interesting to explore the genetic diversity of the binding partners of *PRRT2* in order to decipher whether this lower selective pressure is restricted to *PRRT2* or to other members of the same pathway.

Finally, we showed that whole genome amplification using multiple displacement amplification technology such as GenomiPhi and Repli-G could lead to the appearance of the A217PfsX8 mutation. The genomic region surrounding the mutation is made of 4 guanines and 9 cytosines and seems therefore to be at risk for mutation both *in vivo* and *in vitro*. For this reason, we strongly suggest that *PRRT2* mutation screening should be restricted to native DNA and validate using Sanger sequencing. Interestingly, in the last release of the Exome Variant Server database, at the same location of the A217PfsX8 mutation (hg19, chr16: 29825015), 10% of the *PRRT2* allele contains an additional C (allele A1 in EVS) and 10% have a deletion of a C (allele A2 in EVS), but with very low average sample read depth (N = 10). Given the very high penetrance of the A217PfsX8 mutation in PRRT2-related diseases, it is impossible that 20% of the *PRRT2* alleles carry a frame shift mutation at this position. Especially, since we found the A217PfsX8 mutation in only 1 individual out of 1990 (Allelic frequency = 0.025%). This high frequency of A217PfsX8 mutation in EVS could be related to the DNA amplification step required for whole exome sequencing. Studying the mechanism leading to the A217PfsX8 mutation was beyond the scope of our study, but our observation that a DNA amplification step through RCA could introduce this additional C/G might help in understanding the mechanism leading to A217PfsX8 mutation.

In summary, our study indicates that *PRRT2* mutations do not play a major role in the susceptibility to ASD and confirm that truncating mutations of *PRRT2* are not fully penetrant. We also provide an ascertainment of the genetic diversity of *PRRT2* in worldwide populations as well as important indication on the pitfalls for the mutation screening. All together, these results should help researchers and clinicians to better investigate the role of *PRRT2* in human diseases.

## Supporting Information

Figure S1 Sequence coverage of PRRT2 in Exome Variants Serveur. Total number of sample sequenced (in blue) and average read depth (purple) for *PRRT2* in Exome Variant server. The nucleotide positions are according to PRRT2 from NCBI37/hg19. The average coverage of 10×, 20× and 40× are indicated in red, yellow and green respectively.
(TIF)

Figure S2 Multiple dimension scale (MDS) of the genetic distances between individuals from the Human Genome Diversity Pannel. Insert. MDS restricted to the African populations including the two individuals with ASD carrying the in frame deletion p.A361_P362del (triangles).
(TIF)

Table S1 Cohorts used in this study.
(DOCX)

Table S2 Ethnicity of the cohorts.
(DOCX)

Table S3 PRRT2 primers.
(DOCX)

Table S4 PRRT2 nonsynonymous variants identified in the HGDP.
(DOCX)

Table S5 PRRT2 synonymous variants identified in the HGDP.
(DOCX)

Table S6 PRRT2 coding variants present in dbSNP, Exome variant Server (EVS) and the 1000 genome project (1KG).
(DOCX)

Table S7 Identity by state (IBS) values for the 2 individuals with ASD carrying the mutation p.A361_P362del compared with the IBS from different African populations.
(DOCX)

Table S8 Evolutionary analysis of all genes located within the 16p11.2 deletion.
(DOCX)
