# Correction: Heterogeneous Pattern of Selective Pressure for PRRT2 in Human Populations, but No Association with Autism Spectrum Disorders

**DOI:** 10.1371/journal.pone.0100607

**Published:** 2014-06-12

**Authors:** The PLOS ONE Staff

The legends for Figures 3 and 4 are switched. The authors have provided corrected versions here.

**Figure 3:**
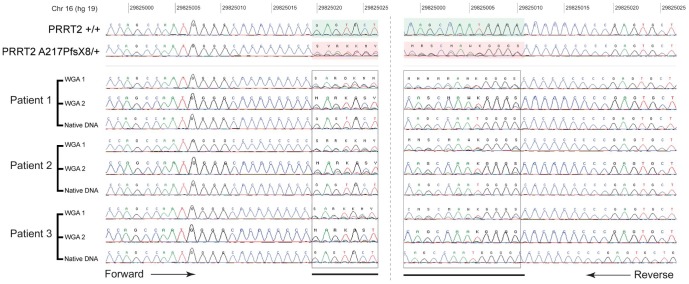
Chromatograms of the *PRRT2* A217PfsX8 mutation before and after whole genome amplification. The chromatograms of *PRRT2* sequence before (Native DNA) and after whole genome amplification of the DNA from three independent patients using two different protocols GenomiPhi DNA Amplification Kit (WGA-1) or Repli-G Whole Genome Amplification kit (WGA-2). The mutation was not present in the native DNA, but was detected after whole genome amplification.

**Figure 4:**
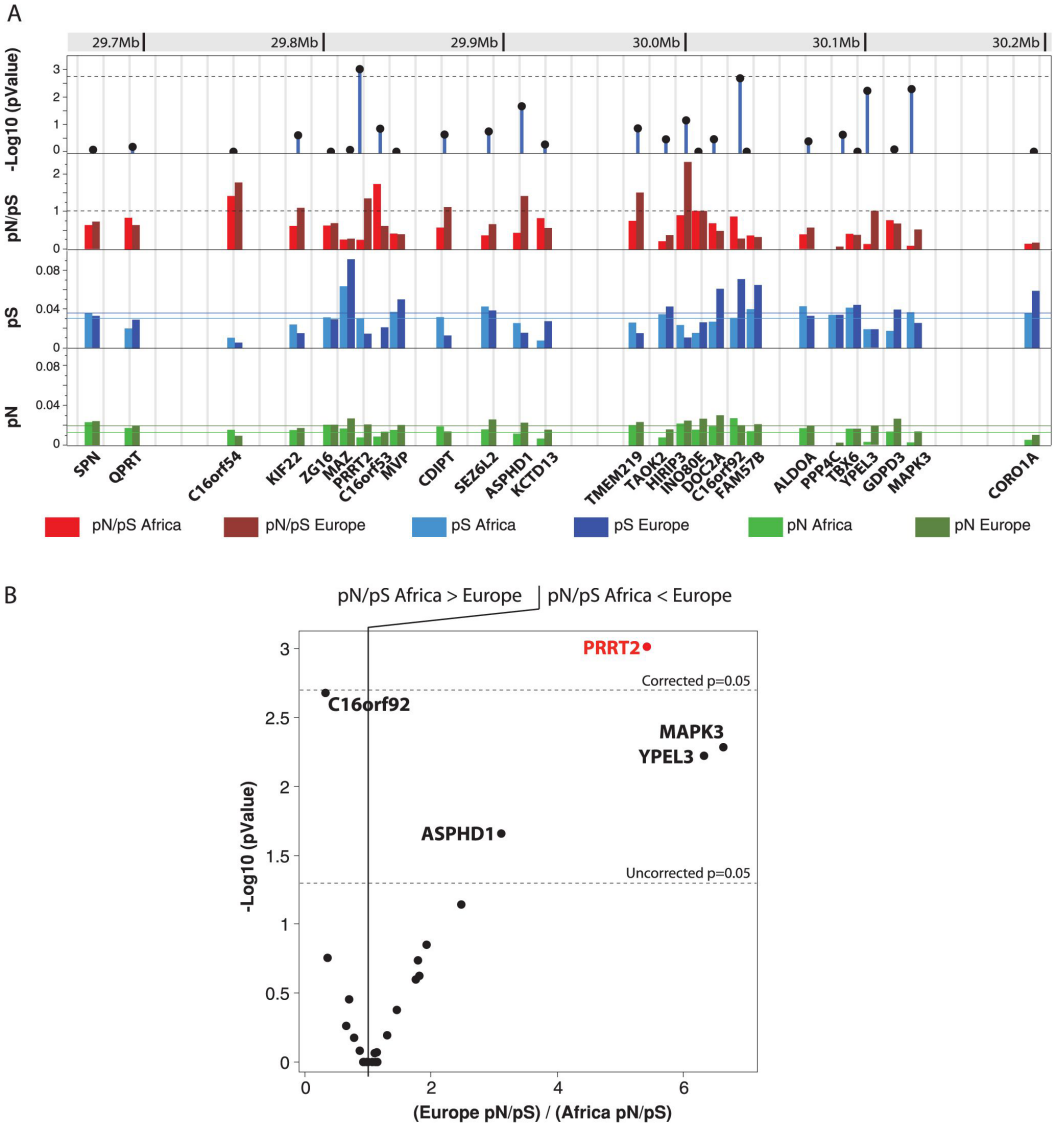
Genetic variability in Europe and Africa for all genes located within the 16p11.2 deletion. A. Nonsynonymous mutations per nonsynonymous sites (pN) and synonymous mutations per synonymous sites (pS) were estimated using the data from 4300 individuals from European ancestry and 2012 individuals from African ancestry available at the Exome Variant Server (http://evs.gs.washington.edu/EVS/). The horizontal lanes correspond to the means of pN and pS for the 27 genes. Difference of pN/pS between Europe and Africa were calculated using a 2-tailed Fisher exact test and the –Log_10_ P value is indicated. B. Plot of the –Log_10_ P values obtained for the difference between the pN/pS ratio in Africa and in Europe in relation the ratio (Europe pN/pS) / (Africa pN/pS).
