# Supplementary figures and images for: Heterogeneous Pattern of Selective Pressure for PRRT2 in Human Populations, but No Association with Autism Spectrum Disorders
Source: PLoS One. 2014 Mar 3;9(3):e88600. doi: 10.1371/journal.pone.0088600 (PMC3940422; doi:10.1371/journal.pone.0088600)

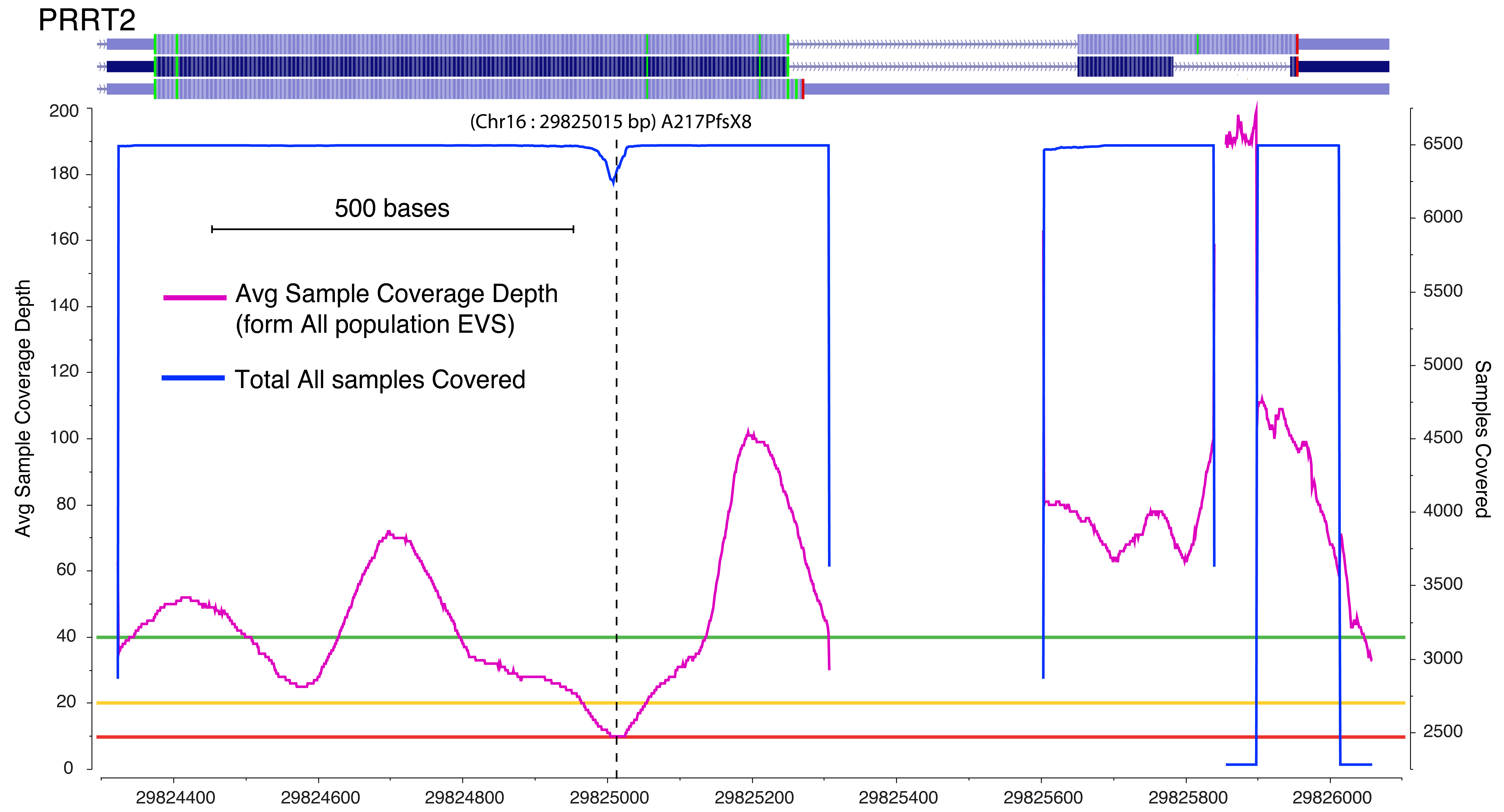

Supplement: Figure S1 — Sequence coverage of PRRT2 in Exome Variants Serveur. Total number of sample sequenced (in blue) and average read depth (purple) for PRRT2 in Exome Variant server. The nucleotide positions are according to PRRT2 from NCBI37/hg19. The average coverage of 10×, 20× and 40× are indicated in red, yellow and green respectively. (TIF) [file pone.0088600.s001.tif]

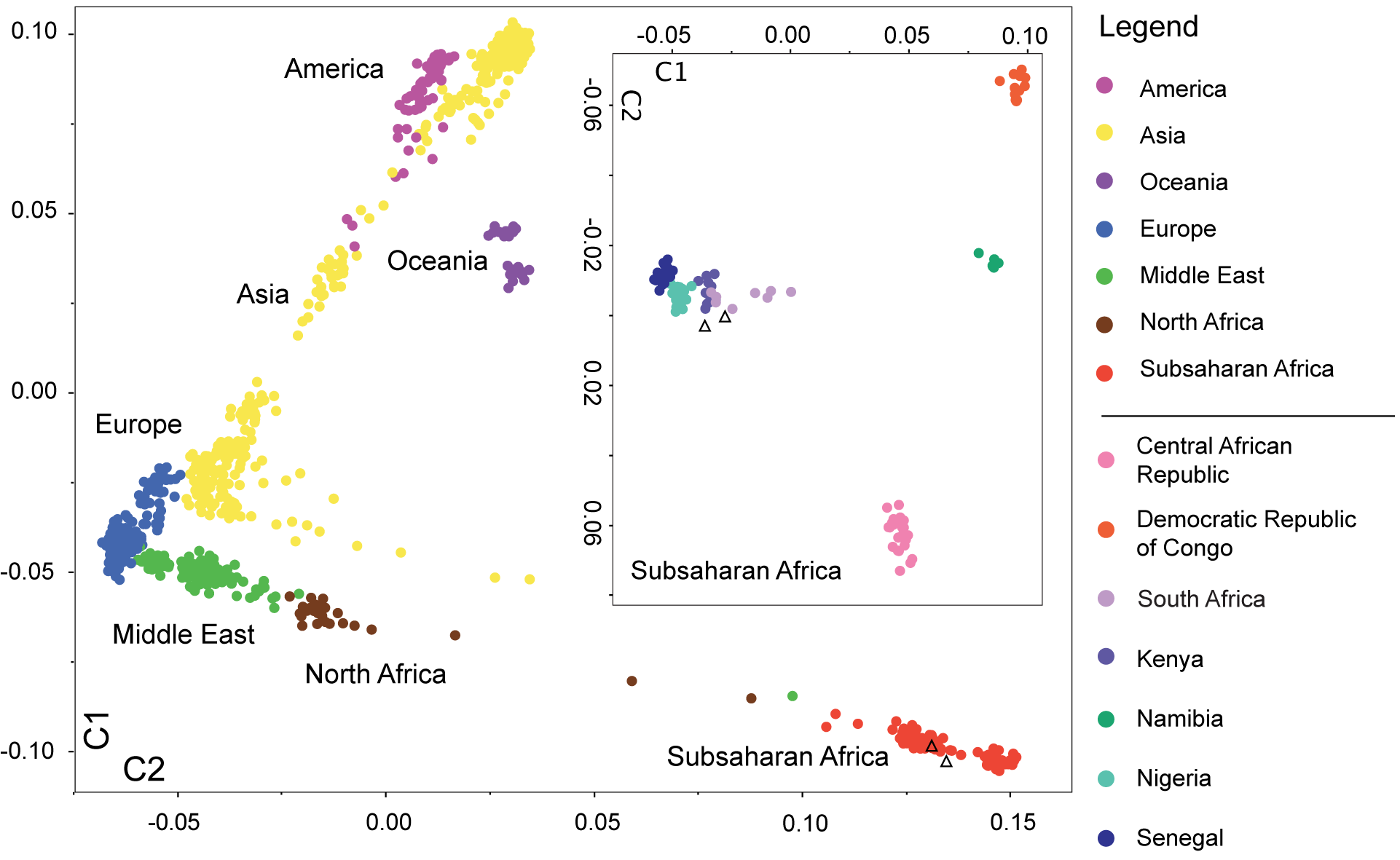

Supplement: Figure S2 — Multiple dimension scale (MDS) of the genetic distances between individuals from the Human Genome Diversity Pannel. Insert. MDS restricted to the African populations including the two individuals with ASD carrying the in frame deletion p.A361_P362del (triangles). (TIF) [file pone.0088600.s002.tif]
