# Supplementary material for: Heterogeneous Pattern of Selective Pressure for PRRT2 in Human Populations, but No Association with Autism Spectrum Disorders
Source: PLoS One. 2014 Mar 3;9(3):e88600. doi: 10.1371/journal.pone.0088600 (PMC3940422; doi:10.1371/journal.pone.0088600)
Supplement: Table S1 — Cohorts used in this study. (DOCX) [file pone.0088600.s003.docx]

# Table S1. Cohorts used in this study

|  | Sex | Asperger syndrome | Atypical autism | Autism | High functioning  Autism | All ASD | Controls | HGDP |
| --- | --- | --- | --- | --- | --- | --- | --- | --- |
| All *PRRT2* coding exons | Female | 8 | 0 | 79 | 2 | 89 | 74 | 332 |
|  | Male | 51 | 2 | 279 | 10 | 342 | 101 | 629 |
|  | All | 59 | 2 | 358 | 12 | 431 | 186 | 961 |
| A217PfsX8 screen only | Female | 9 | 1 | 67 | 1 | 78 | 90 | - |
|  | Male | 17 | 4 | 187 | 9 | 217 | 7 | - |
|  | All | 26 | 5 | 254 | 10 | 295 | 92 | - |
| A217PfsX8 screen All | Female | 17 | 1 | 146 | 3 | 167 | 164 | 332 |
|  | Male | 68 | 6 | 466 | 19 | 559 | 108 | 629 |
|  | All | 85 | 7 | 612 | 22 | 726 | 278 | 961 |
