# Supplementary material for: Heterogeneous Pattern of Selective Pressure for PRRT2 in Human Populations, but No Association with Autism Spectrum Disorders
Source: PLoS One. 2014 Mar 3;9(3):e88600. doi: 10.1371/journal.pone.0088600 (PMC3940422; doi:10.1371/journal.pone.0088600)
Supplement: Table S2 — Ethnicity of the cohorts. (DOCX) [file pone.0088600.s004.docx]

# Table S2. Ethnicity of the cohorts

| Ethnic |  | ASD | |  | | Controls |
| --- | --- | --- | --- | --- | --- | --- |
|  | All *PRRT2* coding exons | | A217PfsX8 screen only | | A217PfsX8 screen All |  |
| Europe | 261 | | 213 | | 474 | 278 |
| Africa | 40 | | 6 | | 46 | - |
| Asia | 13 | | 2 | | 15 | - |
| Mixed origin | 48 | | 12 | | 60 | - |
| Unknown | 69 | | 62 | | 131 | - |
| All | 431 | | 295 | | 726 | 278 |
