# Supplementary material for: Heterogeneous Pattern of Selective Pressure for PRRT2 in Human Populations, but No Association with Autism Spectrum Disorders
Source: PLoS One. 2014 Mar 3;9(3):e88600. doi: 10.1371/journal.pone.0088600 (PMC3940422; doi:10.1371/journal.pone.0088600)
Supplement: Table S3 — PRRT2 primers. (DOCX) [file pone.0088600.s005.docx]

# Table S3. *PRRT2* primers

| Name | Chr | Position start^a^ | Position end | Amplicon size (bp) | Forward | Reverse | Temperature hybridization |
| --- | --- | --- | --- | --- | --- | --- | --- |
| PRRT2 exon 2 start | 16 | 29731680 | 29732220 | 541 | AACCCCAACTTTTCTTCCTCTC | CTCACTTCTGGTTTGGACACTG | 55°C |
| PRRT2 exon 2 middle | 16 | 29732078 | 29732695 | 618 | GCTCCAGAAACCACAGAGACC | GATGGCAAGGATGATGTAGTCC | 64°C |
| PRRT2 exon 2 end | 16 | 29732390 | 29732912 | 523 | TCTGAGAGTGTAGGGGAAAAGC | CTAGGGAGAGGCAAACAAAGG | 59°C |
| PRRT2 exon 3 and 4 | 16 | 29733081 | 29733582 | 502 | TTTCCACCTGATCCCTTCTG | CAGGCTCCCTTGGTCCTTAG | 54°C |

^a^Human Mar. 2006, NCBI36/hg18
