# Supplementary material for: Heterogeneous Pattern of Selective Pressure for PRRT2 in Human Populations, but No Association with Autism Spectrum Disorders
Source: PLoS One. 2014 Mar 3;9(3):e88600. doi: 10.1371/journal.pone.0088600 (PMC3940422; doi:10.1371/journal.pone.0088600)
Supplement: Table S4 — PRRT2 nonsynonymous variants identified in the HGDP. (DOCX) [file pone.0088600.s006.docx]

# Table S4. PRRT2 nonsynonymous variants identified in the HGDP

| Mutation | North Africa  N=29 | Subsaharan Africa  N=108 | Middle East N=135 | Europe N=159 | Asia  N=439 | Oceania N=31 | America N=64 |
| --- | --- | --- | --- | --- | --- | --- | --- |
| S5N | 0 | 0 | 0 | 0 | 1 | 0 | 0 |
| P18T | 0 | 0 | 0 | 0 | 1 | 0 | 0 |
| E23K | 0 | 0 | 0 | 1 | 0 | 0 | 0 |
| P45S | 0 | 2 | 0 | 0 | 0 | 0 | 0 |
| P48R | 0 | 0 | 0 | 0 | 1 | 0 | 0 |
| S115K | 0 | 0 | 1 | 0 | 0 | 0 | 0 |
| P140A | 0 | 0 | 0 | 1 | 43 heterozygotes  8 homozygote | 3 | 10 |
| D147H | 0 | 0 | 0 | 0 | 3 | 0 | 0 |
| T151N | 0 | 0 | 0 | 0 | 1 | 0 | 0 |
| P154S | 0 | 0 | 0 | 0 | 5 | 0 | 0 |
| E180K | 0 | 0 | 1 | 0 | 0 | 0 | 0 |
| A214P | 0 | 0 | 0 | 0 | 5 | 0 | 1 |
| P215R | 0 | 0 | 0 | 0 | 4 | 0 | 0 |
| P215T | 0 | 0 | 0 | 0 | 1 | 0 | 0 |
| P216L | 1 | 0 | 0 | 1 | 1 | 0 | 0 |
| R217Q | 0 | 0 | 0 | 1 | 0 | 0 | 0 |
| G241S | 0 | 0 | 0 | 0 | 0 | 0 | 1 |
| G258E | 0 | 0 | 0 | 0 | 2 | 0 | 0 |
| G258R | 0 | 0 | 0 | 0 | 0 | 4 heterozygotes  1 homozygote | 0 |
| A272V | 0 | 0 | 0 | 0 | 1 | 0 | 0 |
| R311Q | 0 | 0 | 0 | 0 | 1 | 0 | 0 |
