# Supplementary material for: Heterogeneous Pattern of Selective Pressure for PRRT2 in Human Populations, but No Association with Autism Spectrum Disorders
Source: PLoS One. 2014 Mar 3;9(3):e88600. doi: 10.1371/journal.pone.0088600 (PMC3940422; doi:10.1371/journal.pone.0088600)
Supplement: Table S5 — PRRT2 synonymous variants identified in the HGDP. (DOCX) [file pone.0088600.s007.docx]

# Table S5. PRRT2 synonymous variants identified in the HGDP

| Cohort | Region | CEPH ID | Position | Variants | Allele |
| --- | --- | --- | --- | --- | --- |
| ASD Cohort study | Subsaharan Africa | PED-SAL-NGE-1013-005 | 16 : 29765126 | L251L | C/T |
|  |  | PED-SAL-LIO-1157-002 | 16 : 29765126 | L251L | C/T |
|  | Reunion Island | AU-RD-SAJ-209-004 | 16 : 29825914 | P381P | A/C |
|  | Europe | AU-GRE-SEB-132-004 | 16 : 29825026 | R217R | A/G |
| HGDP | Subsaharan Africa | HGDP00460 | 16 : 29825020 | P215P | C/T |
|  |  | HGDP00465 | 16 : 29825020 | P215P | C/T |
|  |  | HGDP01411 | 16 : 29765126 | L251L | C/T |
|  |  | HGDP01419 | 16 : 29765126 | L251L | C/T |
|  |  | HGDP00920 | 16 : 29765126 | L251L | C/T |
|  |  | HGDP00927 | 16 : 29765126 | L251L | T/T |
|  |  | HGDP00932 | 16 : 29825203 | C276C | C/T |
|  | Asia | HGDP00146 | 16 : 29824756 | E127E | G/A |
|  | Middle East | HGDP00632 | 16 : 29765126 | L251L | C/T |
|  | North Africa | HGDP01271 | 16 : 29765126 | L251L | C/T |
