# Supplementary material for: Heterogeneous Pattern of Selective Pressure for PRRT2 in Human Populations, but No Association with Autism Spectrum Disorders
Source: PLoS One. 2014 Mar 3;9(3):e88600. doi: 10.1371/journal.pone.0088600 (PMC3940422; doi:10.1371/journal.pone.0088600)
Supplement: Table S6 — PRRT2 coding variants present in dbSNP, Exome variant Server (EVS) and the 1000 genome project (1KG). (DOCX) [file pone.0088600.s008.docx]

# Table S6. PRRT2 coding variants present in dbSNP, Exome variant Server (EVS) and the 1000 genome project (1KG)

| **NCBI.37**  **Chr:position (bp)** | **rsID** | **Alleles** | **Avg Sample Read Depth** | **AA** | **cDNA Q7Z6L0-2 (bp)** | **Function**  **GVS** | **Polyphen2** | **European American Genotype Count** | **African American Genotype Count** | **HGDP** | **Cohort** | **Source** |
| --- | --- | --- | --- | --- | --- | --- | --- | --- | --- | --- | --- | --- |
| 16:29824442 | rs140383655 | A/G | 49 | E23K | 67 | missense | benign | AA=0/AG=16/GG=4284 | AA=0/AG=3/GG=2194 | HGDP | Cohorts | dbSNP_134 |
| 16:29824448 | unknown | G/C | 49 | P25A | 73 | missense | benign | GG=0/GC=1/CC=4299 | GG=0/GC=0/CC=2197 | - | - | EVS |
| 16:29824455 | unknown | C/A | 49 | H27P | 80 | missense | possibly-damaging | CC=0/CA=1/AA=4299 | CC=0/CA=0/AA=2197 | - | - | EVS |
| 16:29824508 | rs11556732 | T/C | 40 | P45S | 133 | missense | possibly-damaging | TT=0/TC=0/CC=4299 | TT=0/TC=15/CC=2182 | HGDP | - | dbSNP_120 |
| 16:29824599 | rs147004110 | T/C | 27 | P75L | 224 | missense | possibly-damaging | TT=0/TC=2/CC=4297 | TT=0/TC=0/CC=2197 | - | - | dbSNP_134 |
| 16:29824727 | unknown | A/G | 69 | T118A | 352 | missense | benign | AA=0/AG=2/GG=4298 | AA=0/AG=0/GG=2197 | - | - | EVS |
| 16:29824782 | rs11556731 | C/G |  | P136R | 407 | missense | benign |  |  | - | - | dbSNP_137 |
| 16:29824787 | rs79182085 | G/C | 45 | P138A | 412 | missense | benign | GG=0/GC=7/CC=4292 | GG=0/GC=11/CC=2186 | - | - | dbSNP_131 |
| 16:29824805 | rs150501365 | G/C | 37 | P144A | 430 | missense | benign | GG=0/GC=0/CC=4300 | GG=0/GC=2/CC=2195 | - | - | dbSNP_134 |
| 16:29824814 | rs79568162 | G/C |  | D147H | 439 | missense | benign |  |  | HGDP | Cohorts | - |
| 16:29824875 | unknown | T/C | 29 | T167I | 500 | missense | possibly-damaging | TT=0/TC=1/CC=4298 | TT=0/TC=0/CC=2197 | - | - | EVS |
| 16:29824902 | 1KG_16_29824902 | G/A |  | G176E | 527 | missense | benign |  |  | - | - | EVS, 1KG |
| 16:29824955 | rs139516010 | A/G | 23 | E194K | 580 | missense | possibly-damaging | AA=0/AG=1/GG=4299 | AA=0/AG=0/GG=2194 | - | - | dbSNP_134 |
| 16:29825015 | unknown | GCC/G/GC | 10 | - | 640 | Coding Complex | unknown | A1A1=8/A1A2=2/A1R=240/A2A2=21/A2R=501/RR=3204 | A1A1=3/A1A2=1/A1R=149/A2A2=6/A2R=254/RR=1623 | - | Cohorts | EVS |
| 16:29825018 | unknown | T/C | 10 | P215S | 643 | missense | probably-damaging | TT=0/TC=1/CC=4234 | TT=0/TC=0/CC=2145 | - | - | EVS |
| 16:29825019 | unknown | G/C | 10 | P215R | 644 | missense | probably-damaging | GG=0/GC=4/CC=4240 | GG=0/GC=0/CC=2150 | HGDP | - | EVS |
| 16:29825022 | rs76335820 | T/C | 10 | P216L | 647 | missense | probably-damaging | TT=1/TC=60/CC=4195 | TT=0/TC=11/CC=2156 | HGDP | Cohorts | dbSNP_131 |
| 16:29825024 | rs77838305 | C/G |  | R217G | 649 | missense | probably-damaging | probably-damaging |  | - | - | - |
| 16:29825025 | rs75497546 | G/A |  | R217Q | 650 | missense |  |  |  | HGDP | - | - |
| 16:29825054 | unknown | G/C | 20 | R227G | 679 | missense | probably-damaging | GG=0/GC=0/CC=4299 | GG=0/GC=1/CC=2196 | - | - | EVS |
| 16:29825076 | unknown | T/G | 26 | G234V | 701 | missense | probably-damaging | TT=0/TG=1/GG=4299 | TT=0/TG=0/GG=2197 | - | - | EVS |
| 16:29825127 | rs17850747 | T/C |  | L251S | 752 | missense | possibly damaging |  |  | - | - | - |
| 16:29825695 | unknown | C/G | 64 | Q307H | 921 | missense | probably-damaging | CC=0/CG=1/GG=4299 | CC=0/CG=0/GG=2197 | - | - | EVS |
| 16:29825949 | rs144540943 | C/T | 99 | V338A | 1013 | missense-near-splice | possibly-damaging | CC=0/CT=4/TT=4296 | CC=0/CT=0/TT=2197 | - | - | dbSNP_134 |
