# Supplementary material for: Heterogeneous Pattern of Selective Pressure for PRRT2 in Human Populations, but No Association with Autism Spectrum Disorders
Source: PLoS One. 2014 Mar 3;9(3):e88600. doi: 10.1371/journal.pone.0088600 (PMC3940422; doi:10.1371/journal.pone.0088600)
Supplement: Table S7 — Identity by state (IBS) values for the 2 individuals with ASD carrying the mutation p.A361_P362del compared with the IBS from different African populations. (DOCX) [file pone.0088600.s009.docx]

# Table S7: Identity by state (IBS) values for the 2 individuals with ASD carrying the mutation p.A361_P362del compared with the IBS from different African populations.

| Population | AU-RD-158-003 | | | AU-RD-000-001 | | |
| --- | --- | --- | --- | --- | --- | --- |
|  | Min | Average | Max | Min | Average | Max |
| C.A.R | 0.685 | 0.687 | 0.692 | 0.681 | 0.685 | 0.688 |
| D.R. of Congo | 0.678 | 0.680 | 0.683 | 0.675 | 0.676 | 0.677 |
| Kenya | 0.683 | 0.690 | 0.694 | 0.685 | 0.689 | 0.693 |
| Namibia | 0.672 | 0.674 | 0.676 | 0.668 | 0.670 | 0.671 |
| Nigeria | 0.690 | 0.694 | 0.697 | 0.690 | 0.692 | 0.694 |
| Senegal | 0.687 | 0.690 | 0.693 | 0.685 | 0.689 | 0.692 |
| South Africa | 0.689 | 0.693 | 0.695 | 0.689 | 0.692 | 0.696 |
| AU-RD-158-003 | - | - | - | 0.694 | 0.694 | 0.694 |
| AU-RD-000-001 | 0.694 | 0.694 | 0.694 | - | - | - |
