# Supplementary material for: Heterogeneous Pattern of Selective Pressure for PRRT2 in Human Populations, but No Association with Autism Spectrum Disorders
Source: PLoS One. 2014 Mar 3;9(3):e88600. doi: 10.1371/journal.pone.0088600 (PMC3940422; doi:10.1371/journal.pone.0088600)
Supplement: Table S8 — Evolutionary analysis of all genes located within the 16p11.2 deletion. (DOCX) [file pone.0088600.s010.docx]

# Table S8: Evolutionary analysis of all genes located within the 16p11.2 deletion

| Genes | nonsynonymous sites | synonymous sites | African | | | | | Europe | | | | | Pvalue | Odds |
| --- | --- | --- | --- | --- | --- | --- | --- | --- | --- | --- | --- | --- | --- | --- |
|  |  |  | nonsynonymous variants | synonymous variants | pN ^a^ | pS ^a^ | pN/pS | nonsynonymous variants | synonymous variants | pN ^a^ | pS ^a^ | pN/pS |  |  |
| SPN | 870.333 | 332.667 | 20 | 12 | 0.023 | 0.036 | 0.637 | 21 | 11 | 0.024 | 0.033 | 0.73 | 0.85 | 1.138 |
| QPRT | 658.667 | 235.333 | 12 | 5 | 0.018 | 0.021 | 0.857 | 13 | 7 | 0.02 | 0.03 | 0.663 | 0.667 | 0.778 |
| C16orf54 | 497 | 178 | 8 | 2 | 0.016 | 0.011 | 1.432 | 5 | 1 | 0.01 | 0.006 | 1.791 | 1 | 1.146 |
| ZG16 | 381.667 | 122.333 | 6 | 3 | 0.016 | 0.025 | 0.641 | 7 | 2 | 0.018 | 0.016 | 1.122 | 0.252 | 1.757 |
| KIF22 | 1501.333 | 496.667 | 31 | 16 | 0.021 | 0.032 | 0.641 | 32 | 15 | 0.021 | 0.03 | 0.705 | 1 | 1.067 |
| MAZ | 1018.667 | 346.333 | 17 | 22 | 0.017 | 0.064 | 0.263 | 27 | 32 | 0.027 | 0.092 | 0.287 | 0.862 | 1.105 |
| PRRT2 | 763.667 | 259.333 | 6 | 8 | 0.008 | 0.031 | 0.255 | 16 | 4 | 0.021 | 0.015 | 1.358 | 0.00097 | 5.426 |
| C16orf53 | 581 | 184 | 5 | 0 | 0.008 | 0.005 ^b^ | 1.721 ^b^ | 8 | 4 | 0,014 | 0,022 | 0.633 | 0.126 ^b^ | 0.353 |
| MVP | 2015.667 | 666.333 | 33 | 25 | 0.016 | 0.038 | 0.4365 | 43 | 34 | 0.021 | 0.051 | 0.418 | 1 | 0.978 |
| CDIPT | 485.667 | 156.333 | 9 | 5 | 0.019 | 0.032 | 0.58 | 7 | 2 | 0.014 | 0.013 | 1.127 | 0.237 | 1.814 |
| SEZ6L2 | 1916.333 | 645.667 | 31 | 28 | 0.016 | 0.043 | 0.373 | 50 | 25 | 0.026 | 0.039 | 0.674 | 0.183 | 1.792 |
| ASPHD1 | 864.667 | 308.333 | 10 | 8 | 0.012 | 0.026 | 0.446 | 20 | 5 | 0.023 | 0.016 | 1.426 | 0.022 | 3.114 |
| KCTD13 | 741.667 | 248.333 | 5 | 2 | 0.007 | 0.008 | 0.837 | 12 | 7 | 0.016 | 0.028 | 0.574 | 0.547 | 0.653 |
| TMEM219 | 534.333 | 188.667 | 11 | 5 | 0.021 | 0.027 | 0.777 | 13 | 3 | 0.024 | 0.016 | 1.53 | 0.141 | 1.929 |
| TAOK2 | 2399.333 | 750.667 | 18 | 26 | 0.008 | 0.035 | 0.216 | 39 | 32 | 0.016 | 0.043 | 0.381 | 0.354 | 2.24 |
| HIRIP3 | 1302.667 | 368.333 | 29 | 9 | 0.022 | 0.024 | 0.911 | 33 | 4 | 0.025 | 0.011 | 2.333 | 0.072 | 2.48 |
| INO80E | 547 | 188 | 9 | 3 | 0.016 | 0.016 | 1.031 | 15 | 5 | 0.027 | 0.027 | 1.031 | 1 | 1,00 |
| DOC2A | 909 | 294 | 17 | 8 | 0.019 | 0.027 | 0.687 | 27 | 18 | 0.03 | 0.061 | 0.485 | 0.351 | 0.699 |
| C16orf92 | 301 | 98 | 8 | 3 | 0.027 | 0.031 | 0.868 | 6 | 7 | 0.02 | 0.071 | 0.279 | 0.0021 | 0.324 |
| FAM57B | 623.667 | 201.333 | 9 | 8 | 0.014 | 0.04 | 0.363 | 13 | 13 | 0.021 | 0.065 | 0.323 | 1 | 0.923 |
| ALDOA | 954.333 | 302.667 | 16 | 13 | 0.017 | 0.043 | 0.390 | 18 | 10 | 0.019 | 0.033 | 0.571 | 0.419 | 1.456 |
| PPP4C | 717 | 207 | 0 | 7 | 0 | 0.034 | 0,000 | 2 | 7 | 0.003 | 0.034 | 0.082 | 0.241 | ∞ |
| TBX6 | 974.333 | 336.667 | 17 | 14 | 0.017 | 0.042 | 0.419 | 17 | 15 | 0.017 | 0.045 | 0.391 | 1 | 0.933 |
| YPEL3 | 366 | 108 | 1 | 2 | 0.003 | 0.019 | 0.147 | 7 | 2 | 0.019 | 0.019 | 1.033 | 0.006 | 6.333 |
| GDPD3 | 729.333 | 227.667 | 10 | 4 | 0.014 | 0.018 | 0.78 | 20 | 9 | 0.027 | 0.04 | 0.694 | 0.828 | 0.868 |
| MAPK3 | 871 | 269 | 3 | 10 | 0.003 | 0.037 | 0.092 | 12 | 7 | 0.014 | 0.026 | 0.53 | 0.0052 | 6.64 |
| CORO1A | 1048.333 | 337.667 | 6 | 12 | 0.006 | 0.036 | 0.161 | 12 | 20 | 0.011 | 0.059 | 0.193 | 1 | 1.119 |

^a^ Number variants / number site of nonsynonymous or synonymous respectively by gene.  ^b^ Add 1 synonymous variants because there isn’t synonymous variants for to make pN/pS.
